# Identification of Isocitrate Dehydrogenase 2 (IDH2) Mutation in Carotid Body Paraganglioma

**DOI:** 10.3389/fendo.2021.731096

**Published:** 2021-09-20

**Authors:** Fengchao Lang, Abhishek Jha, Leah Meuter, Karel Pacak, Chunzhang Yang

**Affiliations:** ^1^Neuro-Oncology Branch Center for Cancer Research, National Cancer Institute, National Institutes of Health, Bethesda, MD, United States; ^2^Section of Medical Neuroendocrinology, Eunice Kennedy Shriver National Institute of Child Health and Human Development, National Institutes of Health, Bethesda, MD, United States

**Keywords:** paraganglioma, carotid body tumor, PET/CT, isocitrate dehydrogenase 2, IDH2, D-2-hydroxyglutarate

## Abstract

Carotid body paragangliomas (PGLs) are rare neuroendocrine tumors that develop within the adventitia of the medial aspect of the carotid bifurcation. Carotid body PGLs comprise about 65% of head and neck paragangliomas, however, their genetic background remains elusive. In the present study, we report one case of carotid body PGL with a somatic mutation in the gene encoding isocitrate dehydrogenase 2 (IDH2). The missense mutation in IDH2 resulted in R172G amino acid substitution, which exhibits neomorphic activity and production of D-2-hydroxyglutarate.

## Introduction

Paragangliomas (PGLs) are neuroendocrine tumors originating from the chromaffin cells in the neural crest. PGLs can arise near the carotid artery, along nerve pathways in the head and neck. Several studies recently revealed that PGLs are heterogeneous and can be categorized into disease Clusters I and II, based on their molecular subtypes (1, 2). Cluster I PGLs carry genetic abnormalities in metabolic pathways, such as loss-of-function mutations in the genes that encode the succinate dehydrogenase (*SDHx*) and Von Hippel-Lindau (*VHL*) tumor suppressor protein (3). Our recent studies also found that gain-of-function mutations in the oxygen-sensing domain in hypoxia inducible factor 2 (*EPAS1*) can also be related to the development of Cluster I tumors (4). Functional studies further indicate that Cluster I PGLs share many common molecular signatures that are relevant to disease pathogenesis, including a pseudohypoxia phenotype, metabolic reprogramming, and epigenetic shifts. Cluster II PGLs carry mutations in the kinase signaling pathways, such as genes that encode NF1, RET, and MAX (5).

Isocitrate dehydrogenases (IDHs) are metabolic enzymes that are involved in Krebs cycle metabolism. IDH1 is a cytoplasmic enzyme that converts isocitrate into α-ketoglutarate (α-KG) in an NADP^+^-dependent manner. IDH2 is a mitochondrial enzyme that catalyzes a similar reaction with IDH1. IDH3 is a mitochondrial enzyme that catalyzes irreversible oxidative decarboxylation of isocitrate through an NAD^+^ dependent manner (6, 7). Several pioneering studies revealed that missense mutations in IDH1/2 are frequently identified in several types of human malignancies, including lower grade glioma, acute myeloid leukemia, cholangiocarcinoma, and chondrosarcoma (8–11). Cancer-associated *IDH* mutations result in amino acid substitutions at the catalytic center of the enzyme, which lead to neomorphic activity (change-of-function). The mutant IDHs consume α-KG and NADPH and produce D-2-hydroxyglutarate (D-2-HG) and NADP^+^ (12, 13). Many recent findings have demonstrated that D-2-HG is an oncometabolite, which promotes oncogenesis through metabolic reprogramming, epigenetic shifts, and activation of oncogenic pathways (14, 15). Notably, the glutaminolytic and reductive carboxylation are remarkably impacted by cancer-associated IDH mutations, which results in a distinctive metabolic signature among solid cancers (16). The presence of an *IDH* mutation in PGL is of significant interest. Zhang et al. reported one case of an *IDH* heterozygous mutation accompanied by an *ATRX* mutation in one PGL case (17). The mutation of IDH2 was firstly described in a case of head and neck paraganglioma (18). In the present study, we provided detail histological analysis and ^18^F-FDOPA positron emission tomography (PET) scan about this case. The presence of IDH2-R172G mutation was confirmed by an antibody targeting the mutant enzymes. The R172G mutation results in the production of D-2-HG.

## Material and Method

### Tumor Specimen

The tumor specimen and the patient were previously reported (18). A right carotid body tumor was surgically resected for histology, genetics, and biochemistry analysis.

### Whole Exome Sequencing and Data Analysis

Genomic DNA was extracted from tumor tissue and patient blood using DNeasy Blood & Tissue kit (QIAGEN). Library preparation and exome enrichment were conducted using SureSelect V7 kit (Agilent). The sequencing was performed on a NovaSeq S4-300 chip. The resulting FASTQ data was aligned to the human reference genome for variant annotation.

### Sanger Sequencing

Sanger sequencing was performed as previously described (19). Genomic DNA was amplified through polymerase chain reaction (PCR) using Herculase II Fusion DNA polymerase (Agilent). The amplicon was purified using Monarch PCR & DNA Cleanup Kit (New England Biolabs, Inc.) and sent for Sanger sequencing (Eurofin). The primers used for PCR and sequencing were: F: 5’-CAG AGA CAA GAG GAT GGC TAG G -3’ and R: 5’-TGT AAA ACG ACG GCC AGT GTC TGG CTG TGT TGT TGC TTG-3’. Sequence analysis was performed using Geneious Pro software (version 11.1.4).

### Immunohistochemistry

Immunohistochemistry was performed as previously described. Tumor tissues were fixed and sectioned to 10-micron slices. The slices were labeled with primary antibodies and probed with Vectastain ABC kit (Vector laboratories). The slices were then exposed to 3,3′-Diaminobenzidine and mounted in Permount mounting medium (Fisher Chemical). The samples were then visualized by light microscopy (Olympus BX-43). The primary antibody used in this study was anti-IDH1/2 Mutant (R132/R172), clone MsMab-1 (1:200, MilliPore).

### Mutagenesis and Establishment of Stable Cell Line

Mutagenesis was conducted as previously described (20). The pCMV6-IDH2 wild type plasmid (Origene) was used as a template. The coding sequence of IDH2 was transferred to pLenti-C-myc-DDK-IRES-puro plasmid (Origene). The mutagenesis reaction was performed using a Quikchange Mutagenesis Lightning kit (Agilent). The sequence of the mutant plasmid was confirmed by sequencing the entire coding region of IDH2. The plasmid was then used for lentivirus packaging. The hpheo-1 cells were kindly provided by Dr. Hans Ghayee (University of Florida). The hpheo-1 cells were infected with lentivirus with IDH2 coding sequence and selected by puromycin. The mutagenesis primers used were: 5’- CCA TGG GCG TGC CCG CCA ATG GTG ATG -3’; 5’- CAT CAC CAT TGG CGG GCA CGC CCA TGG-3’.

### Western Blot

The tissue specimens or cell pellets were lysed with Radioimmunoprecipitation assay buffer (RIPA buffer, Thermo Fisher) and quantified using the DC Protein Assay (Bio-Rad). Protein samples were resolved on 4-12% Bis-tris gel (Thermo Fisher) and transferred to PVDF membrane (MilliPore). The membranes were labeled with primary antibodies and visualized through chemiluminescence kit (Bio-Rad). The primary antibodies used in this study include anti-IDH1/2 Mutant (R132/R172), clone MsMab-1 (1:2,000, MilliPore); Flag (1:5,000, Origene); and β-actin (1:5,000, Cell Signaling Technology).

### Metabolite Extraction and Measurement

Stock solutions of D- or L-2-hydroxyglutaric acid (D- or L-2-HG) disodium salt (10 mM), and the internal standards (IS) D or L-2-HG -^13^C_5_ disodium salt (5 mM) were prepared in water and stored at -50°C until use. Calibration standard of D- and L-2-HG were prepared by serial dilutions of the stock with 80% methanol. The working IS mixture (1 µM each) was prepared by diluting the stock with 50% methanol. The TSPC (*N*-(p-toluenesulfonyl)-L-phenylalanyl chloride) derivatization reagent was prepared in acetonitrile (1 mg/mL). Tissues were homogenized in 80% methanol (15-75 mg tissue/mL solvent) using the Bead Rupter (Omni International). The supernatant was diluted with 80% methanol if needed to ensure the 2-HG level was within the linear range of the calibration curve. Samples were analyzed in triplicate. TSPC derivatization was carried out according to Cheng et al. (21) with slight modification. In a 0.5 mL micro-centrifuge tube, 30 µL of sample (or standard) were mixed with 10 µL of IS and then vacuum dried. Two µL of pyridine and 100 µL of TSPC were added to the dried samples. After 30 min, the reaction mixture was vacuum dried and reconstituted with 30 µL of 50% acetonitrile for LC/MS/MS. LC was performed with a Prominence 20AD system (Shimadzu). The injection volume was 2 µL. Separation was achieved at 35°C with a 2.1 x 100 mm, 2.7 µm Cortecs C18 column (Waters). Mobile phase A was 5 mM ammonium acetate in water and mobile phase B was methanol. The flow rate was 300 µL/min and peaks were eluted with a 5-min gradient from 25%-40%B. MS/MS was performed with a TSQ Quantiva triple quadrupole mass spectrometer (Thermo Fisher) operating at selected reaction monitoring mode with negative electrospray ionization. The TSPC derivatized peaks were detected using the following m/z precursor > product ions: 2-HG (448 > 318); 2-HG -^13^C_5_ (453 > 318). D- and L-2-HG in the homogenates were determined by a 9-level calibration curve (0.05-25 µM) with linear regression (1/x weighting) using the Thermo Xcalibur software.

### Statistical Analysis

Statistical analysis was performed with GraphPad Prism software (Version 6.01). A student t-test was applied for statistical comparisons. All statistical tests were two-tailed. Results are shown as Mean ± SEM, and P < 0.05 was considered statistically significant.

## Results

### Clinical Characteristics

**Figure 1:**
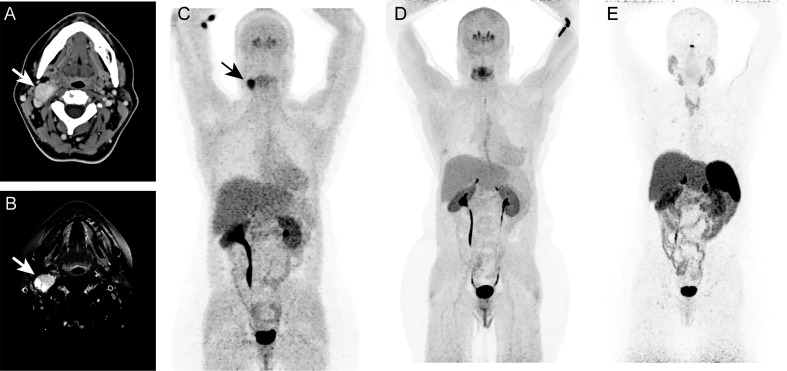
In this figure of a 47-year-old-woman, axial images **(A, B)** of contrast enhanced computed tomography (CT, **A**) and T1 weighted, fat suppressed magnetic resonance imaging **(B)** demonstrates an intensely enhancing 2.7 x 2.0 cm mass (arrows; A, B) at the bifurcation of right common carotid artery causing splaying of right internal and external carotid arteries. The anterior maximum intensity projection (MIP, **C–E**) of ^18^F-fluorodopa (^18^F-FDOPA) positron emission tomography (PET) demonstrates an uptake in the carotid region (arrow, **C**). This mass was identified as a right carotid body paraganglioma. Six years later, follow-up functional PET/CT imaging with ^18^F-FDOPA **(D)** and ^68^Ga-DOTATATE **(E)** shows no evidence of pheochromocytoma/paragangliomas (PHEOs/PGLs). Similarly, whole-body CT (not shown here) imaging and neck MRI (not shown here) were negative for PHEOs/PGLs.

The patient was a 57-year-old female presenting with a history of multinodular goiter and benign thyroid nodules through fine needle aspiration (FNA). The patient also has a family history of thyroid cancer. The patient presented approximately 2.5 x 1.5 cm mass at bifurcation of right common carotid artery with minimal vascularity. Whole-body CT and neck MRI revealed a 2.7 x 2.0 cm mass in the right neck suggestive of carotid body PGL (**Figures 1A, B**). The mass was also positive on ^18^F-fluorodopa (^18^F-FDOPA) PET/CT scan (**Figure 1C**). There was no evidence of any other pheochromocytomas (PHEOs) or PGLs on the aforementioned scans. A 2.8 x 1.8 x 1.1 cm tumor mass was surgically resected, and the operative findings and histopathology confirmed right carotid body PGL. Immunohistochemical studies also showed positivity for chromogranin A and synaptophysin. Six years after surgery, based on follow-up imaging with whole-body ^18^F-FDOPA (**Figure 1D**) and ^68^Ga-DOTATATE PET/CT (**Figure 1E**), as well as whole-body CT and neck MRI, there was no evidence of any recurrent or new PGL.

This patient was enrolled under the protocol Diagnosis, Pathophysiology, and Molecular Biology of Pheochromocytoma and Paraganglioma (ClinicalTrials.gov Identifier: NCT00004847), which was approved by the *Eunice Kennedy Shriver* National Institutes of Child Health and Human Development and Institutional Review Board. Written informed consent was obtained from the patient.

### Expression of IDH2 Mutant in Carotid Body PGL

**Figure 2:**
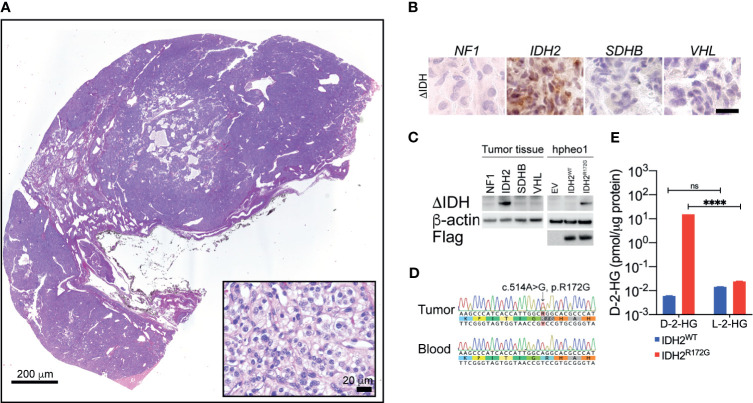
Hematoxylin and eosin staining revealed typical morphology of neuroendocrine tumors **(A)**. Immunohistochemistry showed strong IDH2 mutant expression in the tissue specimen from the index patient **(B)**. Immunoblotting showed the expression of the IDH2 mutant enzyme in cancer tissue. Ectopic expression of IDH2 was used as a positive control **(C)**. Sanger sequencing showed a somatic mutation of the *IDH2* gene in cancer tissue. The variant is not seen in blood DNA **(D)**. Mass spec analysis revealed the presence of high-level D-2-HG in cancer tissue. The level of L-2-HG was found not significantly changed. A non-*IDH*-mutated tumor specimen was used as a control **(E)**. ****p < 0.0001; ns, no significance.

Histopathological examination confirmed the typical PGL morphology (**Figure 2A**). The tumor tissue from the patient exhibited strong immunopositivity for the IDH2 mutant enzyme (**Figure 2B**). The IDH2 mutant immunopositivity was not seen in PGLs with other genetic backgrounds, such as *NF2*, *SDHx* or *VHL*. Further, we examined the expression of IDH2 mutant through immunoblotting. The IDH2 mutant was expressed in the protein lysate from the patient, but not in PGLs with other genetic backgrounds as described above (**Figure 2C**). The specificity of the IDH2 mutant antibody was confirmed by testing the ectopic expression of the IDH2 R172G variant in hPheo1 cell (**Figure 2C**).

### Genetic Analysis

Whole exome sequencing was performed based on DNA specimens from carotid body PGL and patient blood. A somatic change in IDH2 was identified at the location of arginine 172 residue. A follow-up Sanger sequencing confirmed the presence of IDH2 c.514A>G variant, which leads to R172G amino acid substitution (**Figure 2D**).

### Quantification of D-2-HG

To confirm the catalytic function of the IDH2 mutant in cancer tissue, we performed the mass spec measurement on D-2-HG in frozen cancer tissue. We identified 15.39 pmol/µg protein, which was significantly higher than in non-IDH-mutant cancer tissue (0.0059 pmol/µg protein, **Figure 2E**, ****p<0.0001). The level of L-2-HG was found not significantly changed between the tested samples (**Figure 2E**, IDH2^WT^: 0.01436 pmol/µg protein, IDH2^R172G^: 0.2407 pmol/µg protein).

## Discussion

In the present study, we described the presence of a somatic *IDH2* mutation in a carotid body PGL (**Figure 1**). Sequencing analysis revealed a heterozygous *IDH1* R172G mutation in tumor tissue, whereas this mutation was absent in germline DNA (**Figure 2D**). Further, we confirmed the presence of an IDH2 mutant enzyme through immunohistochemistry and immunoblotting (**Figures 2B, C**). The *IDH2* mutation exhibited neomorphic activity, as mass spec analysis revealed substantially elevated D-2-HG levels in tumor tissue (**Figure 2E**).

Mutations of *IDH* are highly prevalent genetic abnormalities in human cancers (22). Cancer-associated *IDH* mutations occur in the catalytic center, resulting in changes in the catalytic function, production of D-2-HG, epigenetic reprogramming, and tumorigenesis (23, 24). The identification of an *IDH2* mutation in a carotid body PGL not only broadens the understanding of these mutations in human cancers (particularly PGL), but it also highlights the role of metabolic deficiency in tumorigenesis of these tumors, implying possible therapeutic regimen by targeting the distinctive metabolic signature. Several small molecule inhibitors against IDH mutant enzymes are currently under investigation through clinical studies, which may be helpful for patients with *IDH*-mutated PGLs.

Due to the critical role of *IDH* mutation in several types human malignancies, pharmacological grade inhibitors have been developed to suppress the neomorphic activity of IDH mutant enzymes (25, 26). Several IDH mutant inhibitors are currently examined through clinical studies, such as AG-120 (e.g., NCT02073994 and NCT02074839), AG-221 (e.g., NCT02273739 and NCT03744390), AG-881 (e.g., NCT02481154 and NCT04164901), and BAY1436032 (e.g., NCT02746081 and NCT04603001). The discovery of an *IDH* mutation in a neuroendocrine tumor suggests the possible application of these inhibitors as potential therapeutic regimens for this type of malignancy. Follow up studies are highly encouraged to explore the prevalence of *IDH* mutations in neuroendocrine tumors. In-depth cancer biology studies will also be helpful to reveal the role of *IDH* mutations in the pathogenesis of neuroendocrine tumors.

## Data Availability Statement

The datasets presented in this article are not readily available because they contain patient information and confidential personally identifiable information (PII). Requests to access the datasets should be directed to the corresponding author.

## Ethics Statement

The studies involving human participants were reviewed and approved by Eunice Kennedy Shriver National Institutes of Child Health and Human Development and Institutional Review Board. The patients/participants provided their written informed consent to participate in this study.

## Author Contributions

CY and KP designed the study. FL and AJ performed the experiment and analysis. FL and CY wrote the manuscript. AJ, LM, and KP revised the manuscript. All authors contributed to the article and approved the submitted version.

## Funding

This research was supported by the Intramural Research Program of the Center for Cancer Research, National Cancer Institute, the *Eunice Kennedy Shriver* National Institute of Child Health and Human Development (NICHD) and the Intramural Research Program of the National Cancer Institute (NCI).

## Conflict of Interest

The authors declare that the research was conducted in the absence of any commercial or financial relationships that could be construed as a potential conflict of interest.

## Publisher’s Note

All claims expressed in this article are solely those of the authors and do not necessarily represent those of their affiliated organizations, or those of the publisher, the editors and the reviewers. Any product that may be evaluated in this article, or claim that may be made by its manufacturer, is not guaranteed or endorsed by the publisher.
